# P-270. Effects of Suppressed Susceptibilities on CRE Case Identification in Clinical Settings in Tennessee, 2016–2022

**DOI:** 10.1093/ofid/ofae631.474

**Published:** 2025-01-29

**Authors:** Lauren Daniel, Ashley Gambrell, Dipen Patel, Raquel Villegas, Daniel Muleta

**Affiliations:** Tennessee Department of Health, Nashville, Tennessee; Tennessee Department of Health, Nashville, Tennessee; HAI/AR, Tennessee Department of Health, Nashville, Tennessee; State of Tennessee, Nashville, Tennessee; Tennessee Department of Health, Nashville TN, Antioch, Tennessee

## Abstract

**Background:**

Carbapenem-resistant Enterobacterales (CRE) are multi-drug resistant organisms (MDRO) often spread in healthcare settings and reported as urgent public health threats by the Centers for Disease Control and Prevention (CDC). Antibiotic susceptibility testing (AST) is often performed, but some results get suppressed to encourage antibiotic stewardship. Tennessee participates in the Multi-site Gram-negative Surveillance Initiative (MuGSI) for CREs in Davidson and seven other surrounding counties. We evaluated the effect of suppression on the clinical identification of CREs in Tennessee.

**Methods:**

Cases reported to MuGSI from 2016­–2022 were analyzed for case determination differences based on AST result interpretations between laboratory reports obtained from instrument and laboratory reports in the medical record. A CRE case was defined as *E. coli*, *Enterobacter cloacae complex*, or *Klebsiella species* resistant to one or more carbapenems and isolated from urine or a normally sterile site, from a resident of the catchment area. Doripenem is excluded from the susceptibility panel in most facilities, and thus not included in analysis. Data analysis was performed using SAS Version 9.4

**Results:**

From 2016–2022 there were 570 cases of CRE, 32% male, 68% female and a mean age of 66 years. Among 570 reported cases, 407 (∼71%) were found to meet the case definition in both the reports obtained from laboratory instrument and reports in medical records. The remaining 163 (∼29%) of cases were found to be CRE based on reports from laboratory instrument but had no AST results for the carbapenems for clinicians in the medical record. The most suppressed carbapenem was imipenem (387), followed by ertapenem (148), and meropenem (119).

**Conclusion:**

Approximately 29% of CRE cases in our data had AST result suppressed or a lack of AST results available in the medical record. Although lack of complete AST results does not preclude the clinician being aware of CRE status, it may result in additional difficulty treating a pathogen with few treatment options. Suppression can lead to better antibiotic stewardship, but MDROs often have complex treatment requirements with high morbidity and mortality for patients. More research is needed to determine the value of suppression as it relates to CREs and MDROs.

**Disclosures:**

**All Authors**: No reported disclosures

